# All-Cause Mortality Outcomes of Usage of Cardiac Contractility Modulation in Patients With Dilated Cardiomyopathy Ineligible for Cardiac Re-Synchronization Therapy: An Updated Meta-Analysis of Randomized Controlled Trials

**DOI:** 10.7759/cureus.10627

**Published:** 2020-09-24

**Authors:** Muhammad Nadeem, Ezza Fatima Tariq, Hafiz M Aslam, Yasir Illahi, Rehan Shah

**Affiliations:** 1 Advanced Heart Failure, University of Kentucky College of Medicine, Lexington, USA; 2 Internal Medicine, Nishtar Medical University and Hospital, Multan, PAK; 3 Nephrology, Oklahoma University Health Sciences Center, Oklahoma City, USA; 4 Hematology and Oncology, East Carolina University, Vidant Medical Center, Greenville, USA; 5 Internal Medicine - Rheumatology, St. Francis Medical Center, Trenton, USA

**Keywords:** cardiac contractility modulation device, mortality, heart failure with reduced ejection fraction (hfref)

## Abstract

Introduction

Dilated cardiomyopathy has been associated with remarkably high mortality despite guideline-directed therapy. This study compares the all-cause mortality rate between a cardiac contractility modulation group and a standard therapy group in patients with dilated cardiomyopathy who were monitored via follow-up for 12 weeks or more.

Materials and methods

We conducted a systematic search of Medline (PubMed) and Cochrane Central Register of Controlled Trials for abstracts and fully published studies (from inception to October 2018). We searched for articles comparing cardiac contractility modulation device therapy with standard therapy for patients with dilated cardiomyopathy between September 1, 2018, and October 30, 2018.

Only fully published randomized clinical trials comparing all-cause mortality outcomes of device therapy and standard therapy for patients with dilated cardiomyopathy were included in our meta-analysis. A total of 673 studies were identified. Studies that were systematic reviews or meta-analyses, study designs or protocols, trials on other regimens, wherein medical therapy was not compared, or wherein the primary outcome of mortality was not assessed, were excluded.

Data were abstracted by two independent reviewers. A random-effect model using the Mantel-Haenszel method calculated the weighted risk ratio (RR). Statistical analyses were performed using Review Manager 5.3 (The Nordic Cochrane Centre, The Cochrane Collaboration; Copenhagen). The primary outcome of interest was a comparison of all-cause mortality between the two groups when patients were monitored via follow-up for 12 weeks or more.

Results

Four fully published randomized clinical trials met the inclusion criteria of our analysis. A random-effect model using the Mantel-Haenszel method calculated the weighted RR. Our analysis included a total of 930 patients. The cardiac contractility modulation therapy group showed no significant reduction in all-cause mortality compared to the standard therapy group (RR, 0.63; 95% CI, 0.29-1.35; P = .23). However, the trend was toward device therapy. Tests for statistical heterogeneity did not show any significant heterogeneity (P = .82, I^2^ = 0%).

Conclusions

Cardiac contractility modulation device therapy is not associated with significant all-cause mortality reduction in patients with dilated cardiomyopathy. Our meta-analysis underscores the need for a large randomized controlled trial on the efficacy of cardiac contractility modulation in a population with dilated cardiomyopathy who are ineligible for cardiac resynchronization therapy.

## Introduction

Heart failure with reduced ejection fractions (HFrEF) accounts for 50% of patients with heart failure. The definition of HFrEF has varied, with guidelines of left ventricular (LV) ejection fraction (EF) of < 35%, < 40%, or ≤ 40%. Remarkably high morbidity and mortality are associated with HFrEF despite standard medical and device therapy in the modern era [1]. Cardiac resynchronization therapy (CRT) has become the standard of care for patients with symptomatic heart failure and delayed myocardial activation, indexed by a prolonged QRS duration [2]. CRT improves ventricular contractile strength, quality of life, exercise tolerance, and reduces mortality and hospitalizations. However, it is estimated that less than half of heart failure patients have dyssynchrony [3], and as many as 30% of patients with implants are considered non-responders [4]. Thus, there is a need for alternative device-based treatments for patients with persistent symptoms despite optimal medical therapy.

A new electrical device, the cardiac contractility modulation (CCM) device, was proposed for enhancing ventricular contractility strength independent of the synchrony of myocardial contraction [5,6]. Preclinical studies have shown that CCM can enhance cardiac muscle contractility acutely [5,7] and that normalization of myocardial gene programs, protein phosphorylation, and reverse remodeling is implicated during long-term CCM signal delivery in animal models of heart failure [8].

CCM includes an implantable pulse generator, the Optimizer™ system (Impulse Dynamics Inc., Orangeburg, New York, USA). The pulse generator delivers highly specialized nonexcitatory electric signals to the myocardium in the right ventricular septum during the absolute refractory period. The resulting enhancement in contractility involves changes in cardiomyocyte Ca2+ handling and normalizing of the messenger ribonucleic expression of heart failure (HF)-related genes [9,10]. Several clinical trials have evaluated the use of CCM devices in patients with New York Heart Association (NYHA) classes II-IV, an EF of 25% to 45% and a narrow QRS complex and have shown significant improvement in the exercise tolerance, peak volume of oxygen (pVO2) and quality of life [11-14]. We have conducted one meta-analysis on the outcomes of cardiac contractility modulation device implantation that included three randomized clinical trials. However, another randomized clinical trial was recently published, hence the need for an updated meta-analysis.

## Materials and methods

Search strategy

We conducted a systematic review of the literature and the meta‐analysis of randomized clinical trials according to established methods and standards recommended by the Cochrane Collaboration and the Preferred Reporting Items for Systematic Reviews and Meta‐Analyses (PRISMA) statement [15,16]. We conducted a systematic search of Medline (PubMed) and Cochrane Central Register of Controlled Trials for abstracts and fully published studies (from September 1, 2018 to 30th October 2018). Our search questions included “Cardiac contractility modulation,” “CCM,” “Heart failure with reduced ejection fraction,” and “HFrEF.” Eligibility criteria included: (i) randomized clinical trial including use of a CCM device; (ii) patients with HFrEF; and (iii) follow-up of 12 weeks or more.

Data extraction and quality assessment

Data were abstracted by two reviewers (YI and MN) independently. Only fully published articles were used for the data collection. Fully published randomized controlled trials (RCTs) were assessed for mortality data, and a minimum follow-up period of 12 weeks or more. Data were abstracted onto Microsoft Excel spreadsheets. The quality of the studies was evaluated using the risk of bias assessment table by Review Manager 5.3 (The Nordic Cochrane Centre, The Cochrane Collaboration; Copenhagen). We assessed the design, conduct, and analysis of the trial using a 3-point scale: low risk of bias, high risk of bias, or unclear. Assessment of risk of bias for each trial included the following questions: (i) Was the allocation sequence adequately generated? (ii) Was the allocation adequately concealed? (iii) Was knowledge of the allocated intervention adequately prevented (i.e., blinded) throughout the trial? (iv) Were incomplete outcome data adequately addressed for every outcome? (v) Were the trial reports free from selective outcome reporting? (vi) Was the trial apparently free of other problems that could put it at risk of bias? Publication bias was assessed for the primary outcome of mortality using a funnel plot.

Data analysis

Review Manager 5.3 was used for statistical analyses. Because of the large amount of heterogeneity in the randomized clinical trials, we used a random-effects model to calculate weighted risk ratio (RR) using the Mantel-Haenszel method (95% confidence interval [CI]) of improvement between treatment and control groups were calculated, and Forest plots were constructed). Heterogeneity between studies was assessed using the I^2^ statistic, according to the Cochrane Handbook. Publication bias was assessed for the mortality outcomes using a funnel plot. The primary outcome of interest was the comparison of all-cause mortality between the two groups.

## Results

**Figure 1:**
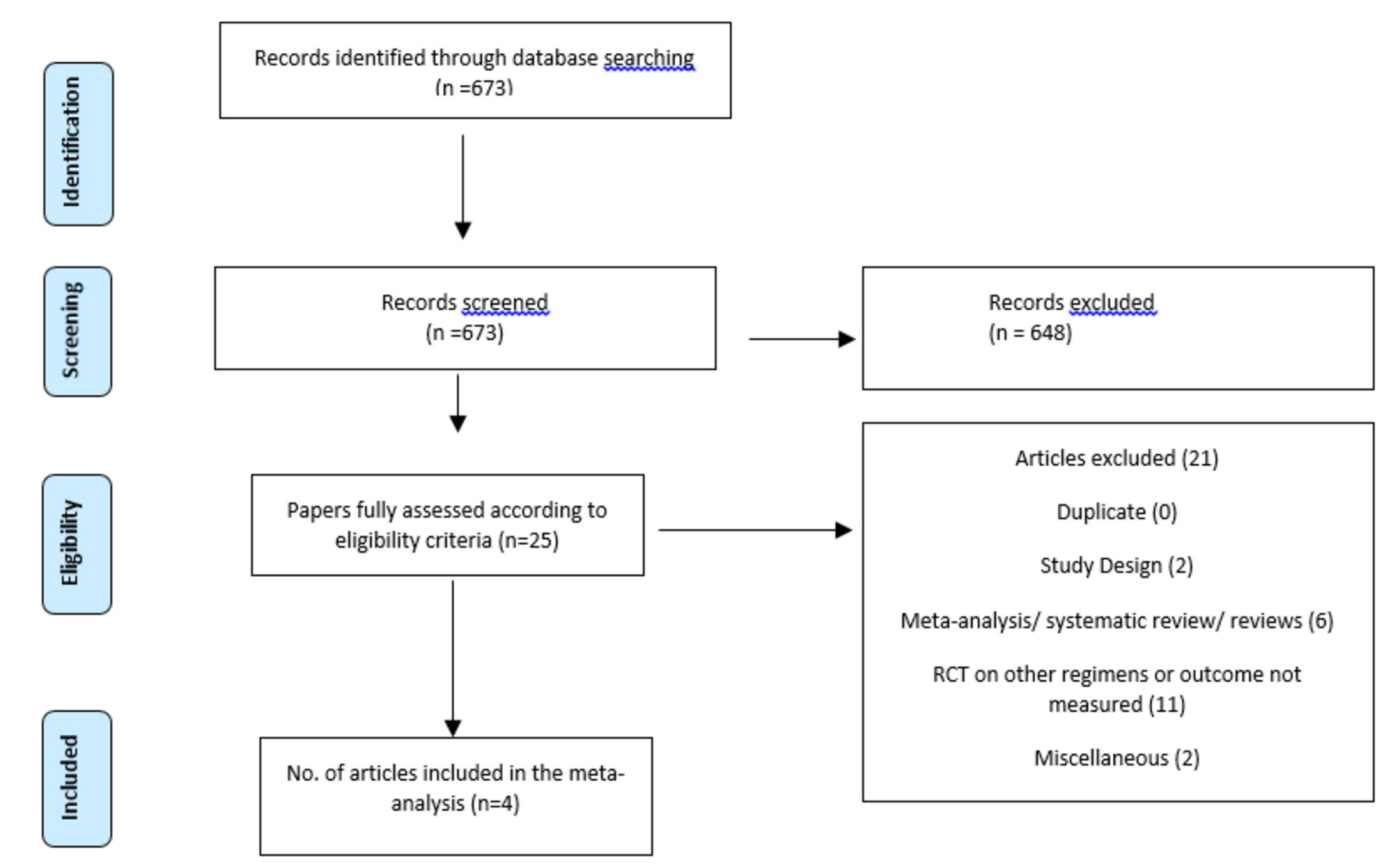
PRISMA diagram

A total of 673 studies were identified. Among these, 648 studies were excluded because they were non-randomized studies. Twenty-five articles were assessed for eligibility criteria. Three fully published randomized clinical trials and one cross-over study were included for the final analysis. Studies that were reviews, systematic reviews or meta-analyses, study designs, rationales or protocols, trials including other outcomes, or featured a follow-up with patients for less than 12 weeks were excluded (PRISMA flow diagram, Figure 1).

Characteristics of included studies

Our inclusion criteria met three randomized clinical trials and one crossover study. Borggrefe et al. was a crossover, randomized, double-blinded study [12]. We used Group 1 as experimental for the first 12-week period and used Group 2 as experimental for the next 12-week period. The mean age ranged between 52 and 63 years among the four studies included. The QRS duration ranged between 101.3 and 119.9 milliseconds among the RCTs. The median EF among the studies was 30.3%. Patients with ischemic cardiomyopathy were more often represented in the RCTs compared to patients with non-ischemic cardiomyopathy. Most of the included patients were from NYHA classes II and III (Table 1).

**Table 1:** Characteristics of included studies Abbreviations: RCT, randomized controlled trial; EF, ejection fraction; NYHA, New York Heart Association; CMP, cardiomyopathy

Study name	Study design	Age (Mean)		QRS duration	EF %	NYHA II	NYHA III	NYHA IV	Ischemic CMP	Non-Ischemic CMP
Abraham et al. [11]	RCT	Experimental	63	103	33	NYHA II & III 86.5%		13.5%	62.2%	37.8%
		Control	63	103.6	33	NYHA II & III 90.7%		9.3%	59.3%	40.7%
Borggerefe et al. [12]	Cross over Study	Group 1	58.9	119.9	29.3	27.5%	72.5%	Nil	63.8%	36.2%
		Group 2	59.9	116.3	29.8	20%	80%	Nil	56%	44%
Kadish et al. [14]	RCT	Experimental	58.09	101.6	25.7	0	91.16%	8.84%	64.7%	35.3%
		Control	58.5	101.5	26.1	0.47%	85.92%	13.62%	66.7%	33.3%
Neelagaru et al. [13]	RCT	Experimental	52.0	109.2	24.9	nil	100%	nil	64%	36%
		Control	59.6	101.3	31.4	nil	96%	nil	67%	33%

**Figure 2:**
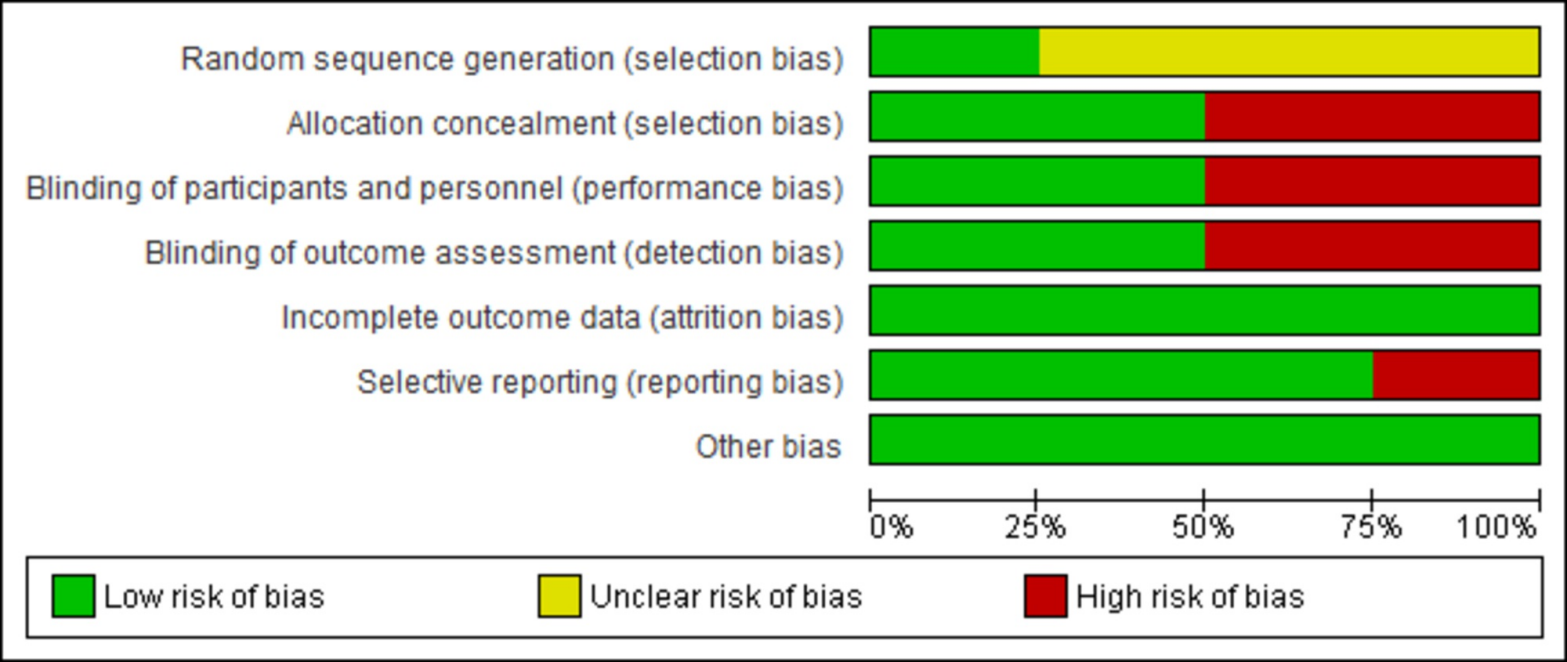
Risk of bias graph

**Figure 3:**
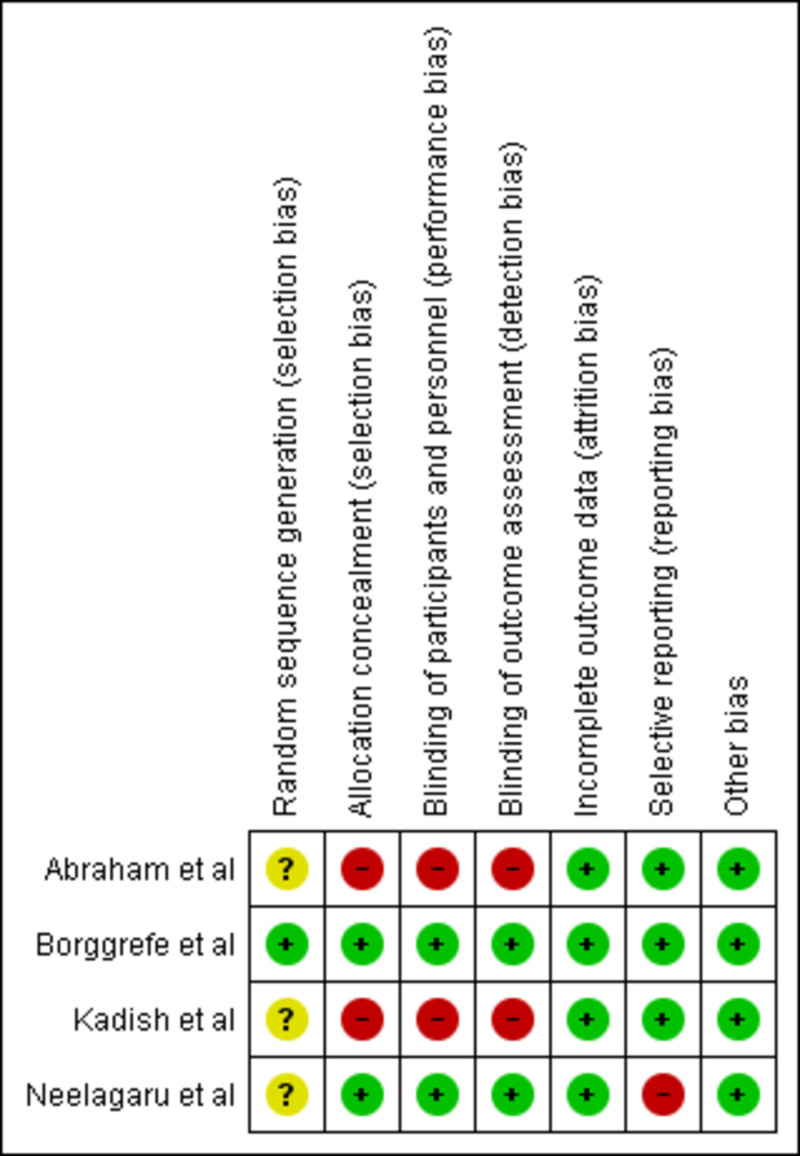
Risk of bias summary

The risk of bias graph and risk of bias summary is shown in Figure 2 and Figure 3, respectively [11-14]. The risk of selection bias (randomization) was mostly unclear because the procedure of randomization was not mentioned. The risk of selection bias (allocation) was high. Abraham et al. and Kadish et al. were unblinded because of the device management protocol and a high probability of violation of the blinding protocol [11,14]. The risk of attrition bias was low for all the included RCTs. Patients recruited by Neelagaru et al. had a significant difference between EF [13], LV end-diastolic diameter (LVEDD), pVO2, and anaerobic threshold. Therefore, the risk of reporting bias was high for the Neelagaru et al. study [13].

Evaluation of the all-cause mortality outcomes

**Figure 4:**
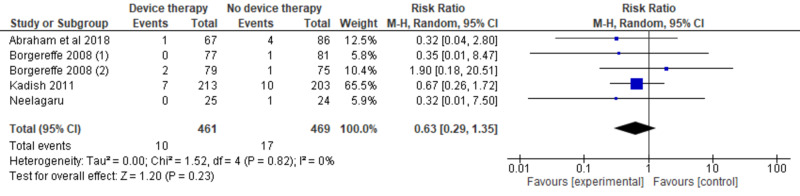
Forest plot of all the included studies indicating all-cause mortality outcomes of the cardiac contractility modulation device in population with HFrEF ineligible for cardiac resynchronization therapy

**Figure 5:**
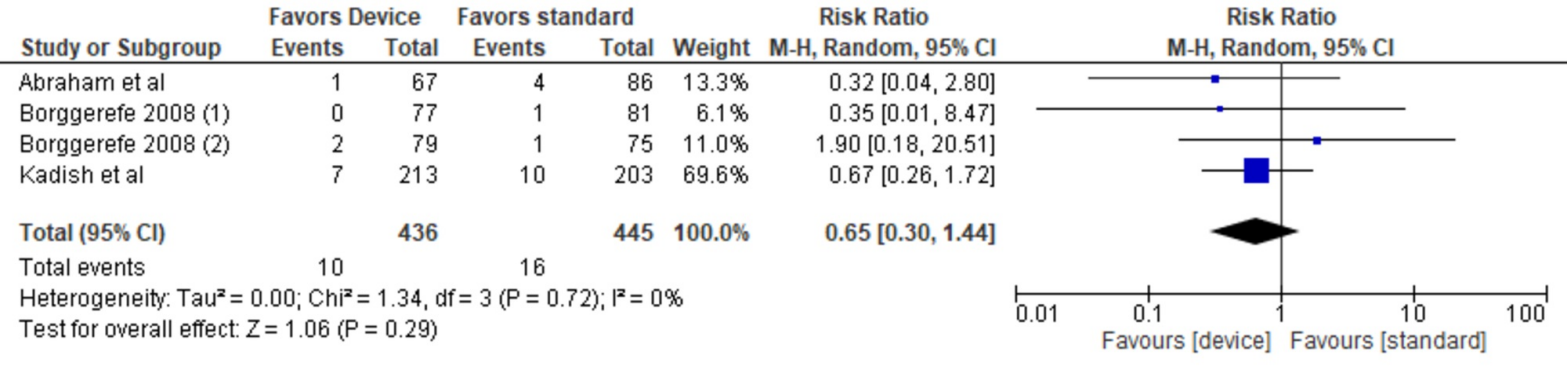
Sensitivity analysis indicating all-cause mortality outcomes of the cardiac contractility modulation device in population with HFrEF ineligible for cardiac resynchronization therapy

**Figure 6:**
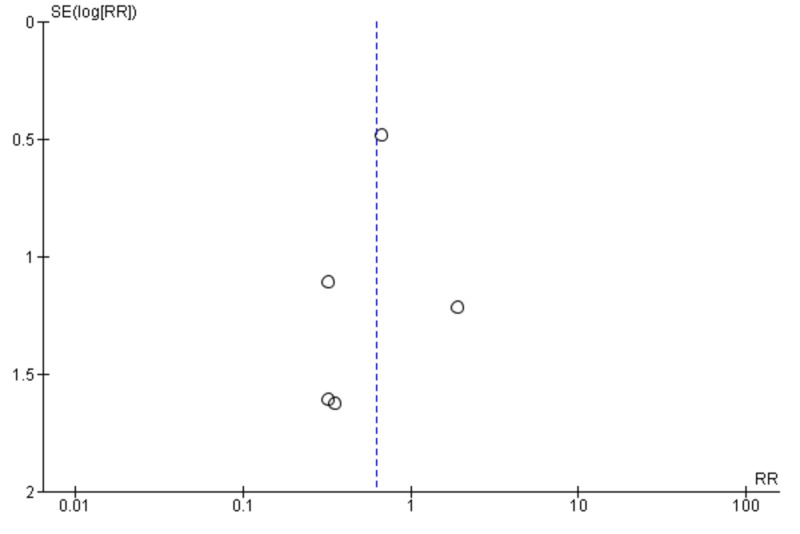
Funnel plot showing distribution of the studies

A random-effect model using the Mantel-Haenszel method calculated the weighted risk ratios (Figure 4) [11-14]. Our analysis included a total of 930 patients. The cardiac contractility modulation therapy group showed no significant reduction in all-cause mortality compared to the standard therapy group (risk ratio [RR], 0.63; 95% CI, 0.29-1.35; P = .23). However, the trend was toward device therapy. Tests for statistical heterogeneity did not show any significant heterogeneity (P = .82, I^2^ = 0%). A visual assessment of a funnel plot of the outcome of mortality did not identify any notable asymmetry suggestive of publication bias (Figure 5). Since the Neelagaru et al. study was at high risk of reporting bias because of a very small sample and a significant difference of EF, LVEDD, pVO2, and the anaerobic threshold between experimental and control groups, we performed a sensitivity analysis of the all-cause mortality outcomes after excluding data of Neelagaru study [13]. However, the sensitivity analysis also showed no significant decrease in all-cause mortality outcomes between the CCM device group and the optimal therapy group (Figure 6) [11,12,14].

## Discussion

HFrEF has been associated with remarkably high mortality despite maximal medical therapy and device therapy [1]. CRT is only indicated in patients with ventricular dyssynchrony represented by a prolonged QRS interval, and 30% of the patients who received a CRT device would not respond [2-4]. The CCM device has shown promising results in animal studies and randomized clinical trials. The CCM has been associated with a significant change in exercise tolerance, pVO2, and improvement of the NYHA class. However, studies showed mixed results when mortality was evaluated [11,12,14]. The previous meta-analysis included three RCTs. Recently, an RCT on the usage of CCM devices in CRT-ineligible patients was published, hence the need for an updated meta-analysis [17].

Our meta-analysis included four RCTs. There was no significant reduction in the all-cause mortality between the CCM device group and the optimal medical therapy group. However, the overall trend was toward device therapy. A sensitivity analysis was performed after excluding Neelagaru et al. [13], and yet there was no significant difference in the mortality outcomes between the device group and the optimal medical therapy group. The previous meta-analysis also evaluated all-cause mortality outcomes between the device therapy group and the optimal therapy group. There was no significant change in the all-cause mortality outcomes (RR, 0.70; 95% CI, 0.47-1.04; P = .078) [17].

In our meta-analysis, selection bias was contributed to by the lack of information provided for the procedure of randomization and allocation concealment. Performance and detection biases were evident in 50% of the studies. Attrition bias was not significant. However, significant differences in the experimental and control groups of the Neelagaru et al. trial must have resulted in significant reporting bias [13].

A randomized clinical trial by Abraham et al. showed that CCM treatment effects were more prominent in patients with an EF > 35% [11]. This finding was also seen in a subset analysis of the FIX-HF-5 (Evaluate Safety and Efficacy of the OPTIMIZER® System in Subjects With Moderate-to-Severe Heart Failure) study [14]. An explanation for this finding cannot be determined with certainty. However, one possible mechanism might include that pacing the right ventricular wall may not be enough for a heart with severely reduced function and severely enlarged size. Therefore, the cohort with better EFs is especially important because of its lack of need for an implantable cardioverter-defibrillator (ICD); thus, implanting a single CCM device could be beneficial [11].

An analysis of the data in the Borggrefe et al. crossover study showed no evidence of a carryover effect from the first to the second phase of the study; the study had a relatively low power to detect such an effect. However, if a carryover were, in fact, present, it would be evident in the group switched from active to sham therapy (i.e., in Group 1), and not in the group switched from sham to active therapy [12]. Therefore, if carryover did exist, it would likely have reduced the estimated treatment benefit and not inflate results. Therefore, the results of the cross-over study likely did not have a reporting bias.

The Borggrefe et al. study was designed on the premise of the Multisite Stimulation in Cardiomyopathies (MUSTIC) trial of CRT [12,18]. The study showed that CCM was of comparable impact on the quality of life and exercise tolerance as CRT. CCM might behave like CRT in terms of measures of improvement, like a change in EF. The Multicenter InSync Randomized Clinical Evaluation (MIRACLE) study of CRT showed only a 1.5% difference increase in EF in the treatment group after three months of follow-up [19]. However, several studies with six months of follow-up showed a more significant increase in the ejection fraction [19,20]. Therefore, longer follow-ups of the current studies and future trials with longer follow-ups are required to evaluate outcomes appropriately [19,20].

There was no increase in the ventricular arrhythmias, ICD shocks, or anti-tachycardia pacing in patients having both ICD and CCM devices [12,13].

## Conclusions

CCM implantation did not show a significant change in the all-cause mortality compared with optimal medical therapy; however, the overall trend was towards device therapy. The cardiac contractility modulation device has been associated with more efficacy at a certain level. It may also behave like CRT when it comes to reverse remodeling and may take longer a follow-up to detect significant changes in the outcomes. CCM has not been associated with an increase in ventricular arrhythmias, ICD shocks, or anti-tachycardia pacing in patients having both ICD and CCM devices.

Our meta-analysis showed that implantation of the cardiac contractility modulation device did not show a significant reduction in the all-cause mortality when compared with an optimal medical therapy group. Our study underscores the need for large scale RCTs for the measurement of all-cause mortality as well as cardiovascular mortality.
